# Machine learning applications in upper gastrointestinal cancer surgery: a systematic review

**DOI:** 10.1007/s00464-022-09516-z

**Published:** 2022-08-11

**Authors:** Mustafa Bektaş, George L. Burchell, H. Jaap Bonjer, Donald L. van der Peet

**Affiliations:** 1 Surgery, Amsterdam UMC Location Vrije Universiteit Amsterdam, De Boelelaan 1117, Amsterdam, The Netherlands; 2 Medical Library, Amsterdam UMC Location Vrije Universiteit Amsterdam, De Boelelaan 1117, Amsterdam, The Netherlands

**Keywords:** Artificial Intelligence, Machine learning, Upper gastrointestinal malignancies, Esophagectomy, Gastrectomy

## Abstract

**Background:**

Machine learning (ML) has seen an increase in application, and is an important element of a digital evolution. The role of ML within upper gastrointestinal surgery for malignancies has not been evaluated properly in the literature. Therefore, this systematic review aims to provide a comprehensive overview of ML applications within upper gastrointestinal surgery for malignancies.

**Methods:**

A systematic search was performed in PubMed, EMBASE, Cochrane, and Web of Science. Studies were only included when they described machine learning in upper gastrointestinal surgery for malignancies. The Cochrane risk-of-bias tool was used to determine the methodological quality of studies. The accuracy and area under the curve were evaluated, representing the predictive performances of ML models.

**Results:**

From a total of 1821 articles, 27 studies met the inclusion criteria. Most studies received a moderate risk-of-bias score. The majority of these studies focused on neural networks (*n* = 9), multiple machine learning (*n* = 8), and random forests (*n* = 3). Remaining studies involved radiomics (*n* = 3), support vector machines (*n* = 3), and decision trees (*n* = 1). Purposes of ML included predominantly prediction of metastasis, detection of risk factors, prediction of survival, and prediction of postoperative complications. Other purposes were predictions of TNM staging, chemotherapy response, tumor resectability, and optimal therapy.

**Conclusions:**

Machine Learning algorithms seem to contribute to the prediction of postoperative complications and the course of disease after upper gastrointestinal surgery for malignancies. However, due to the retrospective character of ML studies, these results require trials or prospective studies to validate this application of ML.

**Graphical abstract:**

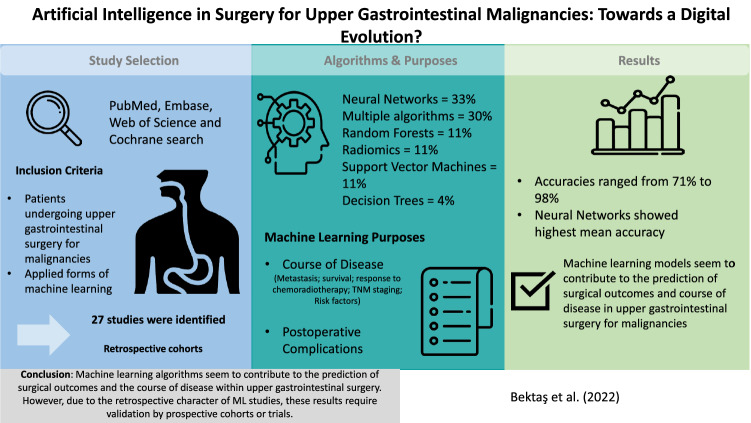

**Supplementary Information:**

The online version contains supplementary material available at 10.1007/s00464-022-09516-z.

Artificial intelligence (AI) has been introduced within healthcare recently, therefore its role has become increasingly important. As the application of AI is escalating, it may be apparent that a digital evolution is ongoing in healthcare [1].

Artificial intelligence is described as the capacity of machines to mimic intelligent human behavior [2]. Artificial intelligence has proven its potential of using large datasets to perform specific tasks, such as image recognition [3]. Machine learning (ML) can be defined as an analytic approach in which models are trained on databases to make predictions on new unseen data [2]. Relevant examples of ML involve support vector machines, decision trees, and gradient boosting. Deep learning is an important subdiscipline of ML, in which multiple datasets are simultaneously included, these datasets are evaluated and modified until the next turn of evaluation. These evaluations are represented as layers and are based on the output of the preceding layer. These processes of evaluation are continued until a final output has been reached. Radiomics are a separate subdiscipline of AI and are able to extract textural features by analyzing medical images [4]. To develop a better understanding, all subdisciplines of AI are depicted in Fig. 1 and explained in Table 1.

**Fig. 1:**
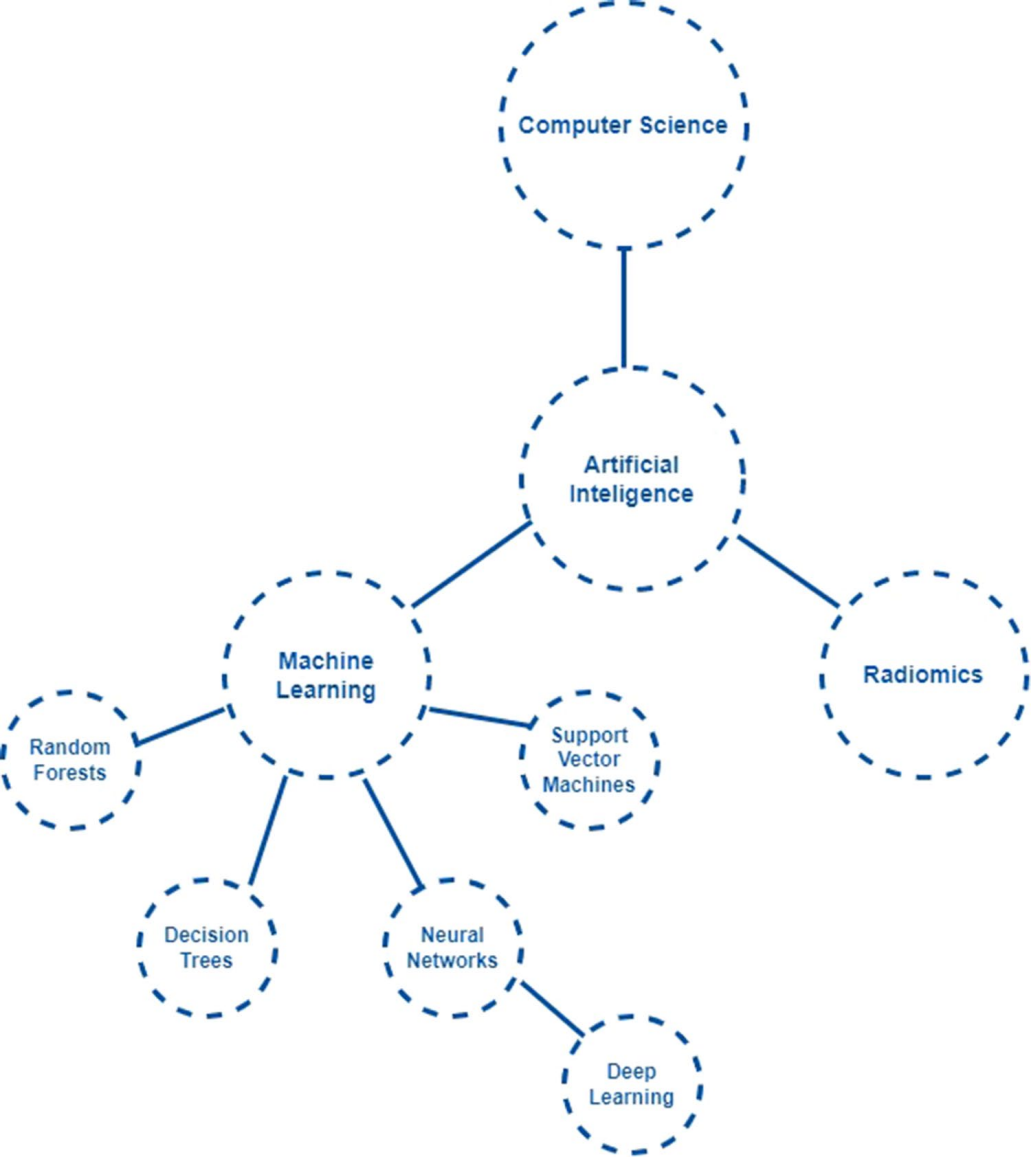
Flowchart of AI subdisciplines

**Table 1:** Definitions of subdisciplines within artificial intelligence

Subdiscipline	Definition
Machine learning (ML)	An ML algorithm includes a calculation process, in which input data is received to perform the desired task along with a specific outcome. After input data are received together with the desired outcomes, this algorithm trains itself, therefore being able to produce the desired outcome from new unknown data [2, 3, 12, 13]
Support vector machine (SVM)	The SVM involves a learning machine, in which input data is mapped into a high-dimensional space, therefore enabling the ability to linearly separate the problem or variable into two groups [14]
Decision tree	A decision tree uses data mining to establish classification systems build on multiple covariates. Each population is classified into segments, represented as branches in the decision tree. This algorithm intends to detect the best model for the data, based on the tree size [15]
Random forest	A random forest model is a collection of decision trees in which each tree produces a separate prediction. In the end, this algorithm combines all predictions to develop an accurate model for regression or classification [16]
Gradient boosting	Gradient boosting models are trained by repeatedly improving inaccuracies of the previous version of the model. This process is continued until a final accurate model is trained [17]
Deep learning	Deep learning uses multiple processing layers to detect certain structures and patterns within large data sets. Each layer provides a concept about the analyzed data, the next concept is then based on the previous one. This process of concept building is continued until the desired output is achieved [2, 3, 18]
Artificial neural networks (ANNs)	ANNs are mathematical models that are based on especially non-linear statistical data. These models mimic several human brain processes by using multiple layers for data analysis and pattern recognition. In each layer, features of data are extracted and weights are calculated for these features within each layer. In the end, a final predictive model is developed by using the most important features that have been selected [19]
Convolutional neural networks (CNNs)	CNNs are a particular type of ANNs, instead of using weights on variables, these neural networks use filters. These filters can understand patterns to create an output that connects with the given input [20]
Radiomics	By using radiomics, quantitative features of images are extracted from mostly radiological imaging. This can provide predictive or prognostic associations with medical outcomes [21]

*ML* machine learning, *SVM* support vector machine, *ANN* artificial neural network, *CNN* convolutional neural network

Since the datasets within electronic medical records are expanding, the abilities of humans to analyze data have been exceeded. As a result, the human role is causing problems such as diagnostic errors, inefficiencies in workflow, and inappropriate treatments in the healthcare system [5]. To eliminate these problems, AI is currently being applied due to its great capacity for analyzing large quantities of data and gaining more experience in a relatively shorter amount of time.

Esophageal and gastric cancer have a high prevalence with poor outcomes, keeping the treatment procedure challenging [6–9]. The introduction of multimodality treatments and minimally invasive techniques have resulted in better treatment outcomes [10]. To further minimize postoperative complication rates, AI tools may be helpful. Another important issue concerns the selection of patients who could optimally benefit from neoadjuvant chemoradiotherapy (nCRT) [11]. Artificial intelligence could contribute to upper gastrointestinal surgery by identifying patients with a complete response to nCRT. Such pre-operative differentiations could be performed if large surgical datasets would be analyzed by ML. Eventually, using ML could provide personalized medicine to optimize treatment outcomes after upper gastrointestinal surgery.

Despite the potential benefits of AI, the scope of ML applications in upper gastrointestinal surgery for malignancies is barely described in literature. However, to support ML implementations in daily practice, it is vital to fill this gap to understand the role and progress of ML within upper gastrointestinal surgery properly. Therefore, this systematic review will provide a comprehensive overview of ML applications in upper gastrointestinal surgery for malignancies.

## Materials and methods

### Search strategy

This study was reported in accordance with the *Cochrane Handbook for Systematic Reviews of Interventions* version 6.0 and PRISMA guidelines. A systematic search was performed in the databases: PubMed, Embase.com, Clarivate Analytics/Web of Science Core Collection, and the Wiley/Cochrane Library. The timeframe within the databases was from inception to the 7th of July 2021 and conducted by M.B. and G.L.B. The search included keywords and free text terms for (synonyms of) 'machine learning' combined with (synonyms of) 'digestive system surgical procedures'. A full overview of the search terms per database can be found in the supplementary information (see appendix 1).

### Study selection

In the first step, to avoid missing studies with overlapping content, articles were included when they described ML within general surgery. Afterwards, studies were only approved if they met the following criteria: (1) describing ML in upper gastrointestinal surgery for malignancies, (2) clinical study, (3) conducted on adults. This review focused only on the most commonly used ML algorithms with adaptive learning abilities, therefore regression models have been excluded as these have been considered traditional statistical approaches. Articles were excluded if they: (1) did not describe the use of ML, (2) the involvement of upper gastrointestinal surgery and malignancies was absent, (3) were not written in English, (4) were publications reporting on reviews, histological analysis, study abstracts, conference proceedings, book chapters, editorials, errata, letters, notes, surveys or tombstones. No peculiar study design was chosen as an inclusion criterium. The title and abstract screening was independently performed by two reviewers (M.B. and G.L.B.) according to the inclusion and exclusion criteria. Studies were included for full-text evaluation when both reviewers agreed on inclusion. Disagreements were solved by discussions between two reviewers, resulting in consensus.

### Quality assessment and risk of bias

The methodological quality of the included randomized controlled trials was assessed using the Revised Cochrane risk-of-bias tool for randomized trials [22]. This tool determines the overall risk of bias based on five bias domains: the randomization process, deviations from intended interventions, missing outcome data, measurement of outcomes, and selection of reported results. The ROBINS-I assessment tool was used to determine the methodological quality of non-randomized studies [23], which determines the overall risk of bias based on seven bias domains; confounding, participant selection, intervention classification, deviations from intended interventions, missing outcome data, measurement of outcomes, and selection of reported results.

### Data synthesis and outcome assessment

After the full-text assessment, the following data from included studies were independently extracted by two reviewers (M.B. and G.L.B.): first author, year, country, number of patients, mean age, study design, carcinoma type, surgical procedure, type of ML, purpose of ML, outcome measurements, and predictive performance. Subsequently, studies were categorized based on surgical procedures and ML subdisciplines, and results of ML use were described within these categories. To summarize the results of studies in quantitative data, the mean accuracy (ACC) and area under the curve (AUC) were calculated as a representation of predictive performances. Conflicts among reviewers were solved by consensus.

## Results

**Fig. 2:**
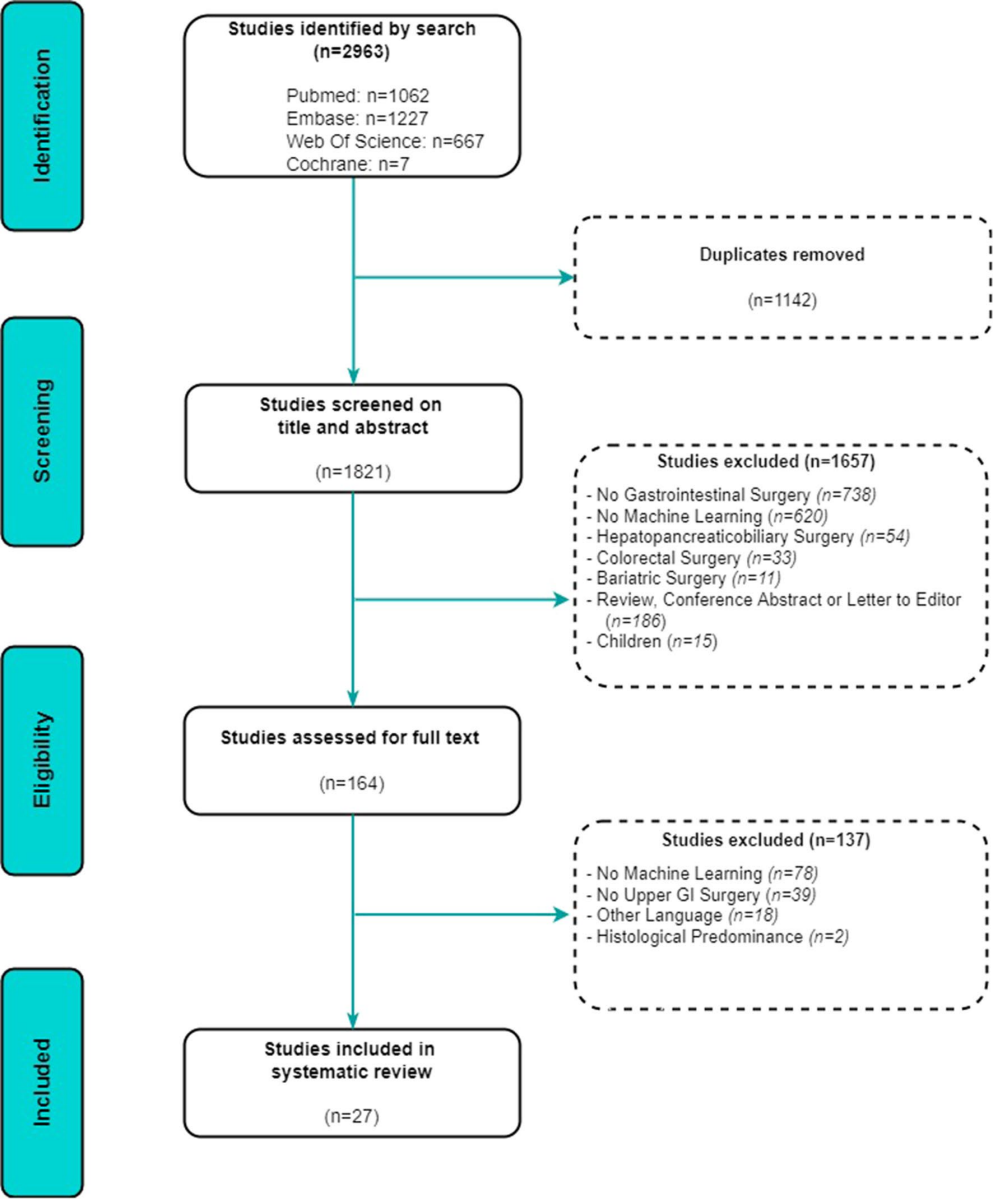
Flowchart of selected articles according to the PRISMA guidelines

The search strategy provided a total of 1821 studies after the removal of duplicates (Fig. 2). Therefore, 1821 studies were screened for eligibility based on the title and abstract screening. Afterwards, 164 studies remained for the full-text assessment, resulting in the inclusion of 27 studies.

In all studies, various ML models have been applied within upper gastrointestinal surgery. The majority of these studies focused on neural networks (*n* = 9), multiple machine learning (*n* = 8), and random forests (*n* = 3). Remaining studies involved radiomics (*n* = 3), support vector machines (*n* = 3), and decision trees (*n* = 1). Eleven studies involved esophagectomy procedures, whereas sixteen studies concerned gastrectomy procedures. Purposes of ML included predominantly prediction of metastasis (*n* = 5), detection of risk factors (*n* = 5), prediction of survival (*n* = 5), and prediction of postoperative complications (*n* = 4). Other purposes were predictions of TNM staging (*n* = 2), chemotherapy response (*n* = 2), tumor resectability (*n* = 2), and optimal therapy (*n* = 2).

In most studies, patients were randomly divided into a training set and a test set. Training sets were used for the development of ML models, afterwards test sets were utilized to determine the accuracy of developed models.

An overview of the study characteristics is presented in Table 2.

**Table 2:** General characteristics of included studies

Authors	Year	Country	Patients s	Age (mean)	Study design	Carcinoma type	Surgical procedures	Type of ML	Imaging	ML purpose	Study outcomes	Predictive performances (ACC/AUC)
Shao et al	2019	China	450	64	Retrospective Cohort	Adeno + Squamous	Esophagectomy	Decision Tree	–	Predict anastomotic leak after surgery	Accuracy; AUC	98%/0.95
Bolourani et al	2021	Canada	383	64	Retrospective Cohort	NA	Esophagectomy	Random Forest	–	Identification of risk factors for early readmission	AUC	–/0.74
Rice et al	2017	USA	5806	63	Retrospective Cohort	Adeno + Squamous	Esophagectomy	Random Forest	–	Detect factors associated with lymph node metastasis	Predictive Probability (%)	NA
Rice et al	2019	USA	13,320	62	Retrospective Cohort	Adeno + Squamous	Esophagectomy	Random Forest	–	Detect optimal therapy for survival	Predictive Probability (%)	NA
Chen et al	2020	China	733	63	Retrospective Cohort	Squamous	Esophagectomy	Neural Networks	–	Predict lymph node metastasis	Accuracy	91%/–
Liu et al	2020	China	523	64	Retrospective Cohort	Squamous	Esophagectomy	Neural Networks	PET + CT	Predict N staging	AUC	–/0.85
Mofidi et al	2006	UK	216	64	Retrospective Cohort	Adeno + Squamous	Esophagectomy	Neural Networks	–	Predict 1 and 3 years disease-free survival after surgery	Accuracy	92%/–
Rishi et al	2021	USA	68	65	Retrospective Cohort	Adeno	Esophagectomy	Radiomics	PET + CT	Predict response to neoadjuvant chemo in esophageal cancer	Accuracy; AUC	77%/0.87
Ou et al	2019	China	591	60	Retrospective Cohort	Squamous	Esophagectomy	Multiple Machine Learning	CT	Predict resectability of esophageal SCC	Accuracy; AUC	NA
Rahman et al	2020	UK	812	64	Retrospective Cohort	Adeno	Esophagectomy	Multiple Machine Learning	–	Predict early recurrence in esophageal adenocarcinoma	AUC	–/0.80
Wang et al	2021	China	153	NA	Retrospective Cohort	Squamous	Esophagectomy	Multiple Machine Learning	CT	Predict overall survival after surgery	C-index	NA
Dai et al	2021	China	78	54	Retrospective Cohort	NA	Gastrectomy	SVM	MRI	Classification of tumor vs healthy tissue	Accuracy	87%/–
Liu et al	2019	China	557	61	Retrospective Cohort	NA	Gastrectomy	SVM	CT	Predict indication D1 vs D2 lymphadenectomy	AUC	–/0.94
Lu et al	2019	China	321	62	Retrospective Cohort	NA	Gastrectomy	SVM	–	Predict postoperative complications after surgery	Accuracy	78%/-
Bollschweiler et al	2004	Germany	135	65	Retrospective Cohort	Adeno	Gastrectomy	Neural Networks	–	Predict lymph node metastasis	Accuracy	93%/–
Jiang et al	2021	China	1978	56	Retrospective Cohort	NA	Gastrectomy	Neural Networks	CT	Predict peritoneal metastasis	AUC	–/0.92
Jin et al	2021	China	1699	57	Retrospective Cohort	Adeno	Gastrectomy	Neural Networks	CT	Predict lymph node metastasis	AUC	–/0.88
Li et al	2020	China	12,108	67	Retrospective Cohort	Adeno	Gastrectomy	Neural Networks	–	Predict 5-year survival after surgery	AUC	–/0.84
Oh et al	2018	Korea	1243	58	Retrospective Cohort	Adeno	Gastrectomy	Neural Networks	–	Predict survival after surgery	AUC	–/0.81
Zhu et al	2013	China	289	63	Retrospective Cohort	Adeno	Gastrectomy	Neural Networks	–	Determine risk factors for gastric cancer	AUC	–/0.89
Chen et al	2021	China	221	57	Retrospective Cohort	Adeno	Gastrectomy	Radiomics	CT	Predict cancer response to chemotherapy	AUC	–/0.74
Li et al	2019	China	554	50	Retrospective Cohort	Adeno	Gastrectomy	Radiomics	CT	Detect predictors of advanced gastric cancer	AUC	–/0.77
Akcay et al	2020	Turkey	75	60	Retrospective Cohort	Adeno	Gastrectomy	Multiple Machine Learning	–	Predict overall survival, metastasis and peritoneal recurrence after surgery	AUC	NA
Celik et al	2019	Turkey	198	57	Retrospective Cohort	NA	Gastrectomy	Multiple Machine Learning	–	Predict anastomotic leak after surgery	Sensitivity; specificity	NA
Feng et al	2019	China	490	62	Retrospective Cohort	NA	Gastrectomy	Multiple Machine Learning	CT	Predict lymph node metastasis	AUC	71%/0.76
Huang et al	2020	China	233	63	Retrospective Cohort	NA	Gastrectomy	Multiple Machine Learning	–	Predict risk factors and diagnosis for lymph node metastasis	Accuracy	76%/–
Qiao et al	2021	China	80	61	Retrospective Cohort	Adeno	Gastrectomy	Multiple Machine Learning	MRI	Predict TNM staging	AUC	NA

*ACC* accuracy, *AUC* area under the curve, *NA* not applicable

### Methodological quality assessment

All included articles were retrospective cohort studies, therefore only the ROBINS-I assessment tool was applied (Table 3). Due to the nature of ML, domains such as bias due to confounding and bias in outcome measurements received low risk-of-bias scores. However, because of the retrospective study design of these studies, moderate risk-of-bias scores were given for bias in the intervention classification domain.

**Table 3:** Methodological quality assessment of the non-randomized studies, according to the ROBINS-I assessment tool

Studies	Bias due to confounding	Bias in participant selection	Bias in intervention classification	Bias due to deviations from intended interventions	Missing data	Bias in outcomes measurements	Bias in reported results	Overall risk of bias
Bolourani et al	Low	Moderate	Moderate	Low	Moderate	Low	Low	Moderate
Chen et al	Low	Low	Moderate	Low	Low	Low	Low	Moderate
Liu et al	Low	Low	Moderate	Low	Low	Low	Low	Moderate
Mofidi et al	Low	Moderate	Moderate	Low	Low	Low	Low	Moderate
Ou et al	Low	Low	Moderate	Low	Low	Low	Low	Moderate
Rahman et al	Low	Low	Moderate	Low	Serious	Low	Low	Serious
Rice et al	Low	Low	Moderate	Low	Moderate	Low	Low	Moderate
Rice et al	Low	Low	Moderate	Low	Moderate	Low	Low	Moderate
Rishi et al	Low	Low	Moderate	Low	Low	Low	Low	Moderate
Shao et al	Low	Low	Moderate	Low	Low	Low	Low	Moderate
Wang et al	Low	Moderate	Moderate	Low	Low	Low	Low	Moderate
Akcay et al	Low	Low	Moderate	Low	Low	Low	Low	Moderate
Bollschweiler et al	Low	Low	Moderate	Low	Low	Low	Low	Moderate
Celik et al	Low	Low	Moderate	Low	Low	Low	Low	Moderate
Chen et al	Low	Low	Moderate	Low	Low	Low	Low	Moderate
Dai et al	Low	Low	Moderate	Low	Low	Low	Low	Moderate
Feng et al	Low	Low	Moderate	Low	Low	Low	Low	Moderate
Huang et al	Low	Moderate	Moderate	Low	Low	Low	Low	Moderate
Jiang et al	Low	Low	Moderate	Low	Low	Low	Low	Moderate
Jin et al	Low	Low	Moderate	Low	Low	Low	Low	Moderate
Li et al	Low	Low	Moderate	Low	Low	Low	Low	Moderate
Li et al	Low	Moderate	Moderate	Low	Low	Low	Low	Moderate
Liu et al	Low	Low	Moderate	Low	Low	Low	Low	Moderate
Lu et al	Low	Low	Moderate	Low	Moderate	Low	Low	Moderate
Oh et al	Low	Low	Moderate	Low	Low	Low	Low	Moderate
Qiao et al	Low	Low	Moderate	Low	Low	Low	Low	Moderate
Zhu et al	Low	Low	Moderate	Low	Low	Low	Low	Moderate

### Esophagectomy

For patients undergoing esophagectomy for malignant esophageal cancer, the following ML models were used: decision trees (*n* = 1), random forests (*n* = 3), neural networks (*n* = 3), radiomics (*n* = 1), and multiple machine learning (*n* = 3).

### Decision tree

Shao et al. developed a decision tree model to predict the occurrence of anastomotic leakage after surgery in patients with esophageal tumors [24]. During the training phase of the model, univariate analysis indicated that the CRP to albumin ratio, postoperative CRP, lymphocytes, surgical duration, postoperative albumin, pre-operative red blood cells, tumor size, TNM, and ASA score were major predictive factors for anastomotic leakage. This model predicted 55 anastomotic leakage cases with an accuracy of 98% and an AUC of 0.95.

### Random forest

Bolourani et al. trained a random forest model to predict patients with early readmissions in 30 days due to severe complications after esophagectomy [25]. Additionally, the most important risk factors for early readmission were determined. Reintubation, prolonged intubation, tracheostomy, aspiration pneumonitis, pyothorax, anastomotic leak, pneumonia, and acute kidney failure appeared to be the essential risk factors for early readmission. After application, the random forest predicted 383 early readmitted patients with an AUC of 0.74. Furthermore, Rice et al. developed a random forest model to detect essential factors associated with lymph node metastasis in patients with esophageal tumors [26]. This model discovered that the increasing cancer size, increasing depth of cancer invasion, and decreasing cancer differentiation are the most important factors for lymph node metastasis after esophagectomy. Additionally, another random forest model was trained to predict the optimal therapy for patients with esophageal tumors [27]. Therapy options included only esophagectomy and esophagectomy combined with adjuvant chemoradiotherapy or neoadjuvant chemoradiotherapy. Optimal therapy was determined as the treatment with a maximum mean survival time. For all patients that received an esophagectomy procedure only, this therapy was optimal in 61% of the cases. In 20% of the remaining patients, the survival time would have increased by 7% if adjuvant therapy was applied, and another 19% would also have benefitted if neoadjuvant therapy was used.

### Neural networks

Chen et al. developed artificial neural networks (ANNs) to predict lymph node metastasis in patients with T1 esophageal tumors undergoing surgery. Multivariate analysis identified six essential factors for developing lymph node metastasis: alcohol use, tumor size, invasion depth, histologic grade, lymph-vessel invasion, and positive imaging results [28]. The ANN model appeared to have an accuracy of 91% for predicting 133 patients with lymph node metastasis. Another study trained an ANN model to accurately predict *N* staging in patients with T1–T2 esophageal carcinomas that underwent surgery [29]. Univariate analysis indicated that tumor invasion depth, tumor length, tumor differentiation, lymph-vessel invasion, and dysphagia were important factors for predicting positive lymph nodes after surgery. By using this ANN model, 148 patients with N1, 65 patients with N2, and three patients with N3 positive lymph nodes were predicted, demonstrating an AUC of 0.85 for this prediction. Another ANN model was developed by Mofidi et al. to predict 1- and 3- year disease-free survival of patients that underwent surgery for esophageal carcinomas [30]. For the 1-year disease-free survival, the ANN model showed an overall accuracy of 88%, whereas an accuracy of 92% was demonstrated for the 3-year disease-free survival.

### Radiomics

Rishi et al. developed a radiomics model for PET and CT scans to predict the response of esophageal tumors to neoadjuvant chemoradiotherapy [31]. Based on multiple radiomic features, patients with a low radiomic score showed a significant improvement in response to neoadjuvant chemoradiotherapy (nCRT). A complete response to nCRT was predicted in 34 patients with an accuracy of 77% and an AUC of 0.87.

### Multiple machine learning

Ou et al. extracted radiomic features and combined them with multiple ML models to predict the resectability of esophageal carcinomas [32]. The resectability of tumors was defined according to the National Comprehensive Cancer Network (NCCN) [33]. A total of 270 patients with resectable carcinomas was predicted by the included ML algorithms. The gradient boosting model showed an accuracy of 79% with an AUC of 0.84. The SVM model indicated an accuracy of 79% with an AUC of 0.82. An accuracy of 69% and an AUC of 0.66 were discovered for the decision tree model. Finally, the random forest model included an accuracy of 67% with an AUC of 0.67 for this prediction. A combined model of the random forest and gradient boosting techniques was developed by Rahman et al. to predict early recurrence of esophageal cancer after surgery [34]. Univariate statistical analysis signified the number of positive lymph nodes and lymphovascular invasion as the most important predictors for early recurrence. Early recurrence after 1 year occurred in a total of 236 patients. Furthermore, for the discriminative ability, this combined model showed an AUC of 0.81 for the interval validation. Application on external databases resulted in an AUC of 0.80. Wang et al. trained a model by combining radiomics with the random forest technique to predict the overall survival of patients that have received surgery for esophageal carcinomas [35]. The index of concordance (C-index) was measured for this model, this index represented the fraction of patients whose survival was correctly predicted from 0 to 1. For this model, the C-index was discovered to be 0.54.

### Gastrectomy

For patients that received a gastrectomy procedure for malignancies, several ML models were applied: SVM (*n* = 3), neural networks (*n* = 6), radiomics (*n* = 2), and multiple machine learning (*n* = 5).

### Support vector machine

Dai et al. developed an SVM model to detect the resectability of gastric cancer [36]. The accuracy of this SVM model was discovered to be 87% in performing a classification between tumor and healthy tissue. Liu et al. trained an SVM model to decide between D1 and D2 lymphadenectomy in patients with gastric cancer [37]. Statistical analysis showed that the T stage, N1 lymph nodes, maximum length of tumor, parenchymal enhancement of tumor, and diameter of lymph node were essential factors for this clinical decision. This SVM model proved an AUC of 0.94 for this classification. Another SVM model was developed to predict postoperative complications after gastrectomy for gastric cancer [38]. The most important clinical features for postoperative complications were discovered to be age, tumor size, comorbidities, hemoglobin, and total protein levels. A total of 100 patients with postoperative complications was predicted by this model with an accuracy of 78%.

### Neural networks

Bollschweiler et al. trained an ANN model with the aim of predicting lymph node metastasis in patients with gastric cancer [39]. An accuracy of 93% was detected for this ANN model in predicting 38 patients with lymph node metastasis. Additionally, Jiang et al. developed an ANN model to predict peritoneal metastasis after surgery [40]. For predicting peritoneal metastasis, tumor type and tumor differentiation appeared to be the key predictors. For this model, an AUC of 0.92 was found for predicting peritoneal metastasis. In another study, a CNN model was trained to predict lymph node metastasis in gastric cancer patients [41]. For lymph node metastasis, tumor size, tumor location, differentiation, depth of invasion, and tumor stage appeared to be the most important predictors. After external validation, an AUC of 0.88 was discovered for this CNN model.

Li et al. developed an ANN model to predict 5-year survival in cohort groups of patients from three different institutes that only underwent a gastrectomy procedure [42]. Essential factors for a 5-year survival were the depth of invasion, lymph node metastasis, age, and tumor size. The AUC for this model appeared to be 0.80, 0.84, and 0.85 in these three groups. Another ANN model was trained to predict 5-year survival after radical gastrectomy [43]. For this model, the AUC was observed to be 0.81 for this prediction. To determine risk factors for the survival of gastric cancer patients, Zhu et al. developed an ANN model for patients that underwent radical gastrectomy [44]. According to this model, tumor stage, radical surgery, serum CA19-9 levels, peritoneal dissemination, and BMI were significant predictors of gastric cancer survival. An AUC of 0.89 was found for this ANN model.

### Radiomics

By training a radiomics model, Chen et al. aimed to predict response to nCRT in patients with advanced gastric cancer [45]. Out of all clinical factors, only tumor differentiation was significant for this response. This model identified 61 patients with complete response to nCRT, and this prediction was performed with an AUC of 0.74. Additionally, Li et al. developed a radiomics model to detect risk factors for advanced gastric cancer [46]. Multivariate analysis indicated that the tumor stage, number of lymph nodes, lymphatic vascular infiltration, and histological grade were key radiomic features for advanced gastric cancer. This radiomics model detected these features with an AUC of 0.87, 0.73, 0.68, and 0.78, respectively.

### Multiple machine learning

Multiple machine learning models were trained by Akcay et al., who aimed to predict overall survival for patients that received chemoradiation therapy after gastrectomy [47]. After multivariate statistical analysis, the most important factors for overall survival were discovered to be the number of removed lymph nodes, the number of metastatic lymph nodes, the lymph node ratio, and the neoadjuvant CT history. The median overall survival was predicted to be 23 months. Gradient boosting showed an AUC of 0.64, whereas the random forest model was observed to have an AUC of 0.59. The SVM model had an AUC of 0.50, and the ANN model had an AUC of 0.45 in predicting the overall survival.

Celik et al. developed an ANN, decision tree, and a random forest model to predict anastomotic leakage after gastrectomy [48]. For postoperative leakage, the random forest and decision tree model both showed a sensitivity of 9% and a specificity of 91%. The ANN model had a sensitivity of 8% and a specificity of 92%. Furthermore, a prediction model was developed based on radiomics and support vector machine techniques. This model was trained to predict lymph node metastasis in gastric cancer patients [49]. 297 patients with lymph node metastasis were identified. External validation showed an AUC of 0.76 and an accuracy of 71% for this model.

Random forest and decision tree models were developed by Huang et al. to predict risk factors and diagnosis for lymph node metastasis in gastric cancer [50]. Random forest showed that tumor size, CT findings, histological grade, hemoglobin, and carcinoembryonic antigen were key factors for the development of gastric lymph node metastasis. The decision tree model was discovered to have an accuracy of 76% for the diagnosis of lymph node metastasis in gastric cancer. Qiao et al. extracted radiomic features and combined them with SVM, decision tree, and random forest algorithms to predict TNM staging in gastric cancer patients [51]. The support vector machine model appeared to have an AUC of 0.79 in predicting TNM staging. The random forest model showed an AUC of 0.78, whereas the decision tree algorithm demonstrated an AUC of 0.63.

## Discussion

From this systematic review, it can be concluded that ML has shown its potential in predicting the course of disease and postoperative complications after upper gastrointestinal surgery for malignancies. Machine learning models have predicted the course of disease with accuracies up to 93% and AUCs up to 0.94, whereas postoperative complications have been predicted with accuracies up to 98% and AUCs up to 0.95.

By using ML, surgeons could be able to pre-operatively identify patients at high risk of postoperative complications. Theoretically, this insight could aid the surgeon in deciding the type of operation and considering prophylactic measures to minimize postoperative complications. For many years, several risk factors for esophagogastric cancer and metastasis have been reported, but as many risk factors are varying from demographical to genetic characteristics, these reports remain controversial [52–54]. Machine learning could be used to select the most important risk factors out of all variables that have been inserted into the model. Studies within this review have already revealed the tumor size, depth of cancer invasion, tumor location, and histologic grade to be essential predictors for the course of disease concerning upper gastrointestinal cancer. Recognizing the most relevant risk factors could be very important for achieving early diagnosis and improving the prognosis of patients with esophagogastric cancer. In addition, ML could also predict response to nCRT in patients with upper gastrointestinal cancer. Based on these predictions, surgeons could reconsider surgical treatment strategies to optimize the treatment outcomes.

Conventional statistics have been applied for many years in surgery. Traditional regression models have been used to predict several aspects concerning the course of disease, such as lymph node metastasis, distant metastasis, survival, and remnant cancer after upper gastrointestinal surgery for malignancies. Predictions were performed with AUCs generally ranging between 0.7 and 0.8 [55–58]. This might be partially overlapping with ML algorithms, as these ranged from 0.7 to 0.98 within this review. However, this review shows that the AUCs of ML algorithms were usually above 0.8, indicating superior predictive capabilities of ML in predicting the course of disease of upper gastrointestinal cancer. Both methods could be used for data analysis and predictions, but there are important differences. Conventional statistics are mainly used to find relationships between usually two variables, whereas ML focuses on recognizing patterns within the data, not being restricted by the number of variables [59]. As patient databases contain a vast amount of clinical variables, ML would be preferred due to its ability to analyze high numbers of variables and complex data.

This review has some limitations. More articles could have been included if more technical databases were searched for ML studies. In addition, due to the presence of inconsistencies in reported accuracies, a meta-analysis could not be performed.

Within this review, it has been discovered that ML models have been only applied retrospectively, therefore prospective studies should be prioritized in the future to gain clinical validation for ML. Gaining this clinical validation could enable patients to receive a personalized treatment plan based on ML predictions. However, to implement ML applications in the field of upper gastrointestinal surgery, a few obstacles need to be overcome. Even though ML models are already applied, clinicians and medical residents still experience difficulties in understanding how the model exactly functions, this is also called “the black box” issue [60]. Additionally, data scientists and engineers have issues with understanding the concepts and interpretations of clinical data, this might impede the development of ML models [61]. To overcome these problems, interdisciplinary collaborations are key to implementing ML applications successfully. To produce high-quality data, data scientists and engineers should be responsible for data preparations, data processing, and feature selections during the training phase. Additionally, clinicians should label clinical data clearly and provide accurate segmentation of esophagogastric cancer on medical images. Clinicians should also focus on feedback from patients to discover additional objectives that could be predicted with ML. However, before these steps, it is essential to agree on study protocols and patient schedules to ensure a systematic and transparent approach to the development of ML models [62]. Furthermore, stakeholders in the hospital should establish strict policies for patient data protection.

## Conclusion

Machine learning has demonstrated accurate predictive capabilities for patients with upper gastrointestinal malignancies undergoing surgery. These capabilities became apparent for short-term outcomes such as prediction of postoperative complications, and long-term outcomes such as prediction of metastasis and survival. However, the current performances of ML are based on retrospective studies only. Therefore, these results require trials and prospective studies to gain clinical validation for ML.

## Supplementary Information

Below is the link to the electronic supplementary material.

Supplementary file1 (DOCX 17 kb)

## Declarations

### Guarantor

M Bektaş.
