# Supplementary material for: Machine learning applications in upper gastrointestinal cancer surgery: a systematic review
Source: Surg Endosc. 2022 Aug 11;37(1):75–89. doi: 10.1007/s00464-022-09516-z (PMC9839827; doi:10.1007/s00464-022-09516-z)
Supplement: Supplementary file 1 — Supplementary file1 (DOCX 17 kb) [file 464_2022_9516_MOESM1_ESM.docx]

**Appendix**

## Table 1: Search strategy in PubMed

| **Search** | **Query** | **Results** |
| --- | --- | --- |
| **#3** | **#1 AND #2** | **1,062** |
| **#2** | **"Digestive System Surgical Procedures"[Mesh] OR "Bariatric Surgery"[Mesh] OR "Laparotomy"[Mesh] OR "Roux-en-Y"[Tiab] OR "Cholecystostom*"[Tiab] OR "Choledochostom*"[Tiab] OR "Gastroenterostom*"[Tiab] OR "Jejunoileal Bypass*"[Tiab] OR "Pancreaticojejunostom*"[Tiab] OR "Peritoneovenous Shunt"[Tiab] OR "Portoenterostom*"[Tiab] OR "Gastric Bypass*"[Tiab] OR "Appendectom*"[Tiab] OR "Cholecystectom*"[Tiab] OR "Sphincterotom*"[Tiab] OR "Colectom*"[Tiab] OR "Cecostom*"[Tiab] OR "Colostom*"[Tiab] OR "Duodenostom*"[Tiab] OR "Ileostom*"[Tiab] OR "Jejunostom*"[Tiab] OR "Esophagectom*"[Tiab] OR "Hemorrhoidectom*"[Tiab] OR "Hepatectom*"[Tiab] OR "Liver Transplant*"[Tiab] OR "Pancreas Transplant*"[Tiab] OR "Pancreatectom*"[Tiab] OR "Pancreaticoduodenectom*"[Tiab] OR "Proctectom*"[Tiab] OR "gastrectom*"[tiab] OR "Gastrostom*"[tiab] OR "Esophagoplast*"[tiab] OR "Esophagostom*"[tiab] OR "Hepatectom*"[tiab]** | **489,138** |
| **#1** | **"Machine Learning"[Mesh] OR "Machine Learning"[Tiab] OR "machine intelligen*"[tiab] OR "machine vision*"[tiab] OR "machine learning"[tiab] OR "transfer learning"[tiab] OR "deep learning"[tiab] OR "neural network*"[tiab] OR "support vector machine*"[tiab] OR "automatic segmentation*"[tiab] OR "Long short term memory"[tiab] OR "LSTM"[tiab] OR "supervised learning"[tiab] OR "unsupervised learning"[tiab] OR "reinforcement learning*"[tiab] OR "hierarchical learning*" [tiab] OR "Image Interpretation*"[tiab] OR "Prediction model*"[tiab] OR "image recognition"[tiab] OR "perceptron"[tiab]** | **154,754** |

## Table 2: Search strategy in Embase.com

| **Search** | **Query** | **Results** |
| --- | --- | --- |
| **#5** | #3 NOT #4 | **1,227** |
| **#4** | #3 AND ('chapter'/it OR 'conference abstract'/it OR 'conference paper'/it OR 'conference review'/it OR 'editorial'/it OR 'erratum'/it OR 'letter'/it OR 'note'/it OR 'short survey'/it OR 'tombstone'/it) | **857** |
| **#3** | #1 AND #2 | **2,084** |
| **#2** | 'gastrointestinal surgery'/exp OR 'laparotomy'/exp OR 'biliary tract surgery'/exp OR (‘Roux-en-Y’ OR ‘Cholecystostom*’ OR ‘Choledochostom*’ OR ‘Gastroenterostom*’ OR ‘Jejunoileal Bypass*’ OR ‘Pancreaticojejunostom*’ OR ‘Peritoneovenous Shunt’ OR ‘Portoenterostom*’ OR ‘Gastric Bypass*’ OR ‘Appendectom*’ OR ‘Cholecystectom*’ OR ‘Sphincterotom*’ OR ‘Colectom*’ OR ‘Cecostom*’ OR ‘Colostom*’ OR ‘Duodenostom*’ OR ‘Ileostom*’ OR ‘Jejunostom*’ OR ‘Esophagectom*’ OR ‘Hemorrhoidectom*’ OR ‘Hepatectom*’ OR ‘Liver Transplant*’ OR ‘Pancreas Transplant*’ OR ‘Pancreatectom*’ OR ‘Pancreaticoduodenectom*’ OR ‘Proctectom*’ OR ‘gastrectom*’ OR ‘Gastrostom*’ OR ‘Esophagoplast*’ OR ‘Esophagostom*’ OR ‘Hepatectom*’):ti,ab,kw | **720,511** |
| #1 | 'machine learning'/exp OR (‘Machine Learning’ OR ‘machine intelligen*’ OR ‘machine vision*’ OR ‘machine learning’ OR ‘transfer learning’ OR ‘deep learning’ OR ‘neural network*’ OR ‘support vector machine*’ OR ‘automatic segmentation*’ OR ‘Long short term memory’ OR ‘LSTM’ OR ‘supervised learning’ OR ‘unsupervised learning’ OR ‘reinforcement learning*’ OR ‘hierarchical learning*’ OR ‘Image Interpretation*’ OR ‘Prediction model*’ OR ‘image recognition’ OR ‘perceptron’):ti,ab,kw | 335,846 |

## Table 3: Search strategy in Clarivate Analytics/Web of Science Core Collection

| **Search** | **Query** | **Results** |
| --- | --- | --- |
| **#4** | #1 AND #2  Refined by: [excluding] DOCUMENT TYPES: ( LETTER OR MEETING ABSTRACT OR EDITORIAL MATERIAL OR CORRECTION ) | **667** |
| **#3** | #1 AND #2 | **747** |
| **#2** | TS=("Roux-en-Y" OR "Cholecystostom*" OR "Choledochostom*" OR "Gastroenterostom*" OR "Jejunoileal Bypass*" OR "Pancreaticojejunostom*" OR "Peritoneovenous Shunt" OR "Portoenterostom*" OR "Gastric Bypass*" OR "Appendectom*" OR "Cholecystectom*" OR "Sphincterotom*" OR "Colectom*" OR "Cecostom*" OR "Colostom*" OR "Duodenostom*" OR "Ileostom*" OR "Jejunostom*" OR "Esophagectom*" OR "Hemorrhoidectom*" OR "Hepatectom*" OR "Liver Transplant*" OR "Pancreas Transplant*" OR "Pancreatectom*" OR "Pancreaticoduodenectom*" OR "Proctectom*" OR “gastrectom*” OR “Gastrostom*” OR “Esophagoplast*” OR “Esophagostom*” OR “Hepatectom*”) | **294,577** |
| **#1** | TS=("Machine Learning" OR “machine intelligen*” OR “machine vision*” OR “machine learning” OR “transfer learning” OR “deep learning” OR “neural network*” OR “support vector machine*” OR “automatic segmentation*” OR “Long short term memory” OR “LSTM” OR “supervised learning” OR “unsupervised learning” OR “reinforcement learning*” OR “hierarchical learning*” OR “Image Interpretation*” OR “Prediction model*” OR “image recognition” OR “perceptron”) | **477,557** |

## Table 3: Search strategy in Wiley/Cochrane Library

| **Search** | **Query** | **Results** |
| --- | --- | --- |
| **#3** | #1 AND #2 | **7** |
| **#2** | ("Roux en Y" OR "Cholecystostom*" OR "Choledochostom*" OR "Gastroenterostom*" OR "Jejunoileal Bypass*" OR "Pancreaticojejunostom*" OR "Peritoneovenous Shunt" OR "Portoenterostom*" OR "Gastric Bypass*" OR "Appendectom*" OR "Cholecystectom*" OR "Sphincterotom*" OR "Colectom*" OR "Cecostom*" OR "Colostom*" OR "Duodenostom*" OR "Ileostom*" OR "Jejunostom*" OR "Esophagectom*" OR "Hemorrhoidectom*" OR "Hepatectom*" OR "Liver Transplant*" OR "Pancreas Transplant*" OR "Pancreatectom*" OR "Pancreaticoduodenectom*" OR "Proctectom*" OR “gastrectom*” OR “Gastrostom*” OR “Esophagoplast*” OR “Esophagostom*” OR “Hepatectom*”):ti,ab,kw | **4,113** |
| **#1** | ("Machine Learning" OR “machine intelligen*” OR “machine vision*” OR “machine learning” OR “transfer learning” OR “deep learning” OR “neural network*” OR “support vector machine*” OR “automatic segmentation*” OR “Long short term memory” OR “LSTM” OR “supervised learning” OR “unsupervised learning” OR “reinforcement learning*” OR “hierarchical learning*” OR “Image Interpretation*” OR “Prediction model*” OR “image recognition” OR “perceptron”):ti,ab,kw | **4,733** |
